# The clinical application of incisional negative pressure wound therapy in severe subcutaneous emphysema: A case series

**DOI:** 10.1016/j.tcr.2024.101026

**Published:** 2024-04-09

**Authors:** Luis Fernandez, Reginald Carl Baptiste, Rebekah Bjorklund, Ala'a Alkhatib, Nesiya Sheriff, Claudia Sanchez, Mary Anne Obst, Rebecca Swindall

**Affiliations:** a Department of Surgery, UT Health East Texas, 1000 S Beckham, Tyler, TX 75701, USA; b Cardiothoracic Surgery, CHRISTUS St. Michael Hospital, 2600 St. Michael Dr., Texarkana, TX 75503, USA; c General Surgery Resident, University of Texas Health Science Center at Tyler, 11937 U.S. Highway 271, Tyler, TX 75708, USA; d University of Texas Medical Branch, 301 University Blvd, Galveston, TX 77555, USA; e Regions Hospital, 640 Jackson St, Saint Paul, MN 55101, USA

**Keywords:** Severe subcutaneous emphysema, High-volume accumulation, Incisional negative pressure wound therapy, Novel clinical application

## Abstract

Severe subcutaneous emphysema (SSE) is the presence of a high-volume accumulation of air in the subcutaneous tissue caused by traumatic injuries, infections, iatrogenic causes, or can also manifest spontaneously. A variety of techniques have been reported, with varying levels of success.

We present a multicenter case series detailing four patients who developed SSE and were treated with Incisional Negative Pressure Wound Therapy (INPWT). All patients significantly improved with the INPWT treatment within 6 to 48 h. Our experience suggests INPWT is a valuable procedure available for treating SSE and recommend prospective randomized studies be conducted to determine targeted patient selection and clinical application of INPWT among the SSE patient population.

## Background

Severe subcutaneous emphysema (SSE) is caused by traumatic injuries, infections, iatrogenic causes, or spontaneous occurrence and can cause patients to experience respiratory distress and severely swollen neck and facial feature [[1], [2], [3], [4], [5]]. Traditional management of SSE involves reducing ventilation pressures and close monitoring [[1], [2], [3], [4]]. Incisional Negative Pressure Wound 3M™ V.A.C.® Therapy (INPWT) has been reported to reduce SSE recovery time and application is straightforward [[1], [2], [3], [4]]. This case series demonstrates SSE treatment with INPWT.

## Case presentations

### Patient 1

A 59-year-old female level 1 trauma patient with past medical history of chronic obstructive pulmonary disease, hypertension, laryngeal cancer and a poor healing wound post tracheostomy, was found on the ground, unresponsive. She was hypotensive, in hypovolemic shock and intubated. Chest X-ray showed evidence of two left rib fractures with a pneumothorax. A left chest tube thoracostomy (CTT) was placed. Follow-up imaging appeared to show residual apical pneumothorax, and a second left CTT was placed.

On examination, the abdomen was soft, non-edematous, ecchymotic extremities with a 4 cm skin tear on the right forearm. The patient was on a ventilator, moving all extremities, responsive with nonverbal cues, had a Glasgow score of 10 T, with no focal deficits. A chart review revealed this was the third time she had cardiorespiratory arrest from hypercarbia. Arterial blood gas showed a PCO2 in the 60s, with a normal pH suggesting chronic hypercarbia. The ventilator settings were adjusted accordingly. The patient was otherwise hemodynamically stable.

**Fig. 1:**
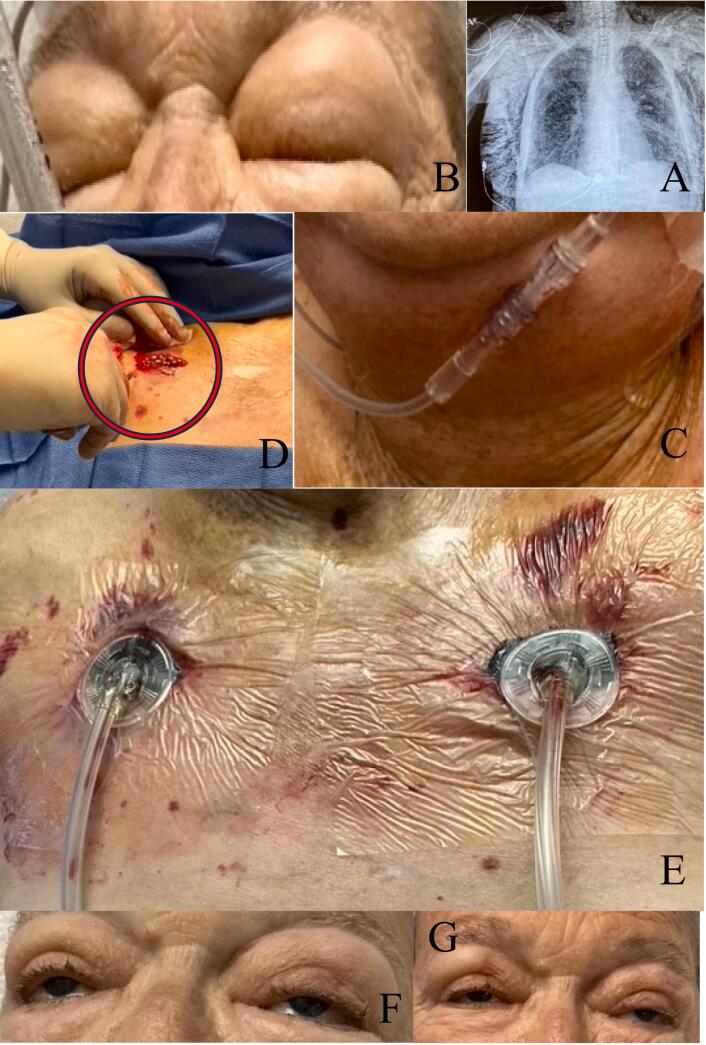
(A–G) Case presentation 1. (A) Follow up chest X-ray 1-hour after initial chest tube thoracostomy placement, (B) Follow up examination: eyes swollen shut, (C) Follow up examination: neck double the size compared to initial exam, (D) Initial incision with bubbling of subcutaneous gas noted [red circle], (E) Bilateral placement of negative pressure wound therapy, (F) Patient after 6 h, (G) Patient after 24 h.

A follow up chest X-ray showed SSE involving the left lateral chest wall [Fig. 1(A)]. A repeat examination the next morning revealed diffuse head-to-toe SSE (Grade V) [4]. The patient could not open her eyes, and her neck was double the size compared to the initial exam [Fig. 1(B,C)]. INPWT was performed at the anterior chest wall (ACW), bilaterally [Fig. 1(D,E)]. The patient tolerated the procedure, symptoms improved within 6 h, and resolved in 24 [Fig. 1(F,G)]. INPWT was discontinued after 4 days, and she was subsequently discharged to a rehab facility.

### Patient 2

A 39-year-old female arrived as a trauma level 1 from an outside hospital after a motor vehicle collision. Diagnostic imaging from the outside hospital revealed multiple pelvic, rib, and spinal fractures, a grade 4 splenic laceration, tachycardia, and bilateral pneumothorax with SSE. Upon arrival, the patient's pulse was in the 140 s, systolic blood pressure was responsive to fluid resuscitation, and bilateral chest tubes were inserted, and embolization of the spleen was performed. The patient was hemodynamically stabilized. Her abdomen was soft with no distension. There were no neurological deficits, and the patient was oriented to person, place, and time. She had no past medical or surgical history, a prescription of omeprazole, and a BMI of 58.06 kg/m^2^.

The patient experienced increasing tachycardia and tachypnea and was placed on BiPAP. Chest tubes were placed bilaterally due to progression of SE and a small apical pneumothorax. On hospital day (HD) 2, her chest tubes were replaced due to the sentinel holes of original chest tubes being in the subcutaneous tissue. During the procedure, a bronchoscopy was performed, and a large mucus plug was removed from the right upper lobe. The patient remained intubated.

**Fig. 2:**
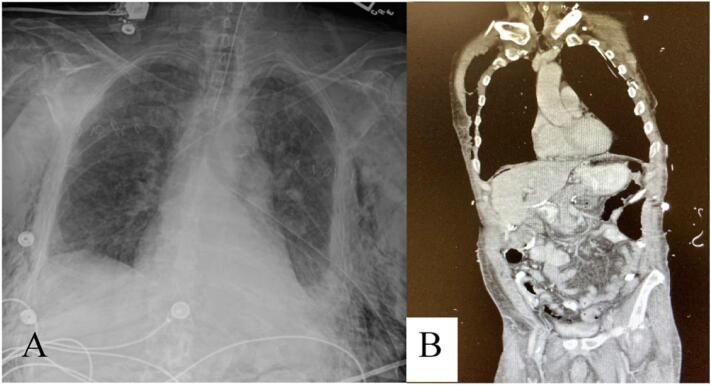
(A–B) Case presentation 2. (A) Chest X-ray HD 4, (B) Progression severe subcutaneous emphysema to the neck and abdomen.

On HD 4, repeat scans demonstrated progression of SSE extending to the neck and abdomen, (Grade V) [4] [Fig. 2(A,B)]. To control the SSE, bilateral, ACW/INPWT was performed. The following morning the patient had significant improvement. The INPWT was removed 4 days after placement, and she was subsequently discharged to a rehab facility.

### Patient 3

A 60-year-old white male with a history of obesity was admitted for pneumonia and right thoracic cavity empyema. Previous medical history included human immunodeficiency virus, with no history of previous infections or complications from the disease. The patient underwent video-assisted thoracoscopic surgery, decortication for empyema and developed a prolonged air leak requiring a chest tube to remain in place and transfer to long-term acute care.

After 8 weeks of ongoing and increased air leakage he was transferred for placement of two endobronchial valves, where he showed signs of improvement. However, 48 h later he was intubated due to hypoxemia and SSE involving his face neck and torso (Grade V) [4].

**Fig. 3:**
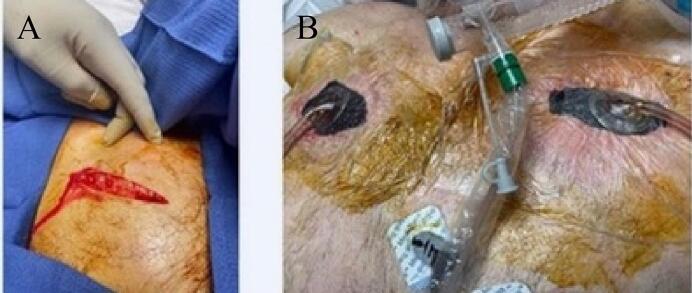
(A–B) Case presentation 3. (A) Anterior chest wall incision, with release of subcutaneous air, (B) Bilateral placement of negative wound pressure therapy.

A chest CT scan showed extensive SSE and pneumomediastinum, with no significant pneumothorax. The ACW/INPWT was applied, the patient tolerated the procedure, and he was subsequently discharged to an inpatient rehabilitation facility on HD 10 [Fig. 3(A,B)].

### Patient 4

A 72-year-old male, intubated, due to hypoxemic acute respiratory failure, was transferred from an outside facility, after falling 12 stairs. The patient had a previous medical history of hypertension, factor V Leiden, prostate cancer, and emphysema. Upon examination, displaced and non-displaced left rib fractures, a trace left pneumothorax and a traumatic brain injury without intracranial hemorrhage were identified. The patient was placed on high flow oxygen.

On HD 2 the pneumothorax increased, and a left CTT was inserted. Chest X-rays identified the CTT was encroaching into the pulmonary parenchyma, this was removed and replaced.

**Fig. 4:**
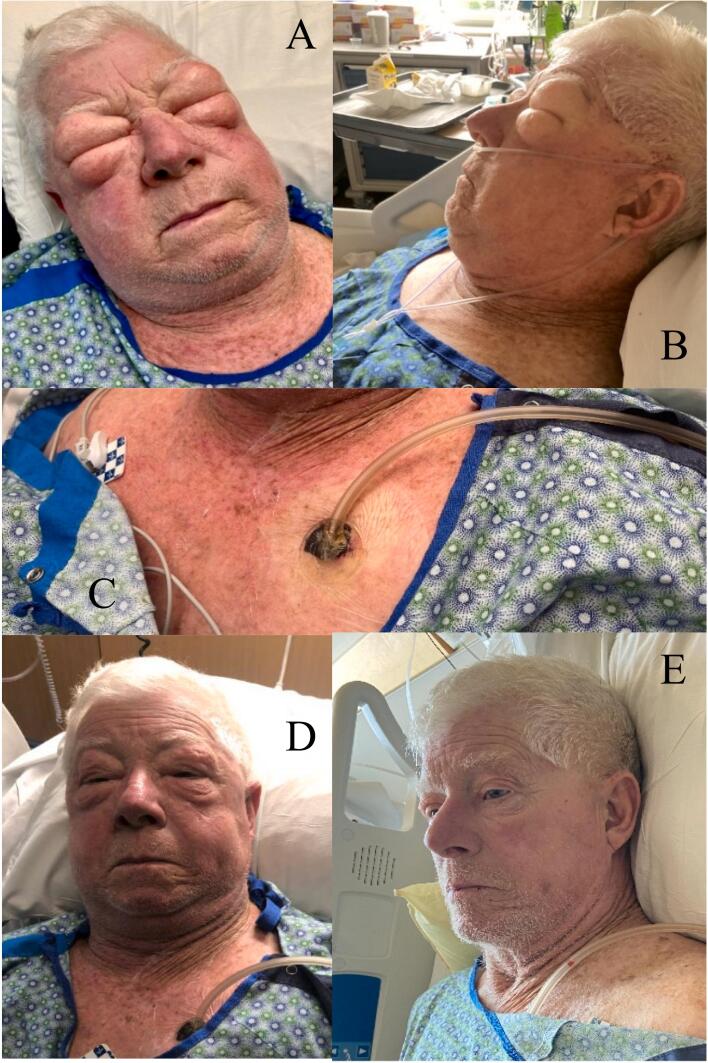
(A–E) Case presentation 4. (A,B) Severe subcutaneous emphysema hospital day 5, (C) Incisional negative wound pressure therapy to the left anterior chest wall, (D) Patient after 24 h, (E) Patient after 48 h.

The patient developed SSE on HD 5 (Grade IV) [4] [Fig. 4(A,B)]. INPWT to the left ACW was performed [Fig. 4(C)]. The patient showed significant improvement at 24 h [Fig. 4(D)], and near total resolution at 48 h [Fig. 4(E)].

## Discussion

Subcutaneous emphysema (SE) may be due to aerodigestive trauma, infections, spontaneous causes, complications of thoracic surgical interventions and iatrogenic injury [[1], [2], [3], [4], [5], [6]]. In most patients SE is nonfatal and self-limited [4,6]. SSE, conversely, can be life-threatening, causing compartment syndrome, restrict expansion of the thorax, tracheal compression, and tissue necrosis [4]. Rapid gaseous expansion may be accelerated with the use of nitrous oxide in conjunction with PPV, leading to increased morbidity and mortality [7].

Standard therapeutic management has limited capabilities in treating SSE due to pneumothorax. Using blowhole incisions alone has demonstrated limited effectiveness as a treatment option and may heighten risk of infection [4,[8], [9], [10]]. Reports on efficacy of subfascial angio catheters also vary and benefit from additional compressive massage [8]. Subcutaneous drainage has demonstrated improvement in SSE, where multiple drainage protocols have been described, also with varying results [9,10]. In all four presented cases, although functioning chest tube thoracostomies were placed, the SSE did not resolve. To manage the complications of SSE more effectively, ACW / INPWT was applied to all patients with rapid improvement of the SSE. In a review of involving SSE and NPWT, identifying the optimal location and technique was inconclusive [1]. Further work towards developing ACW / INPWT treatment protocols in the presence of SSE may prove beneficial.

## Conclusion

Our findings suggest ACW/INPWT is a beneficial, clinical approach in the treatment of SSE. Prospective randomized studies to determine optimal patient selection and clinical application of INPWT for SSE is warranted.

## Limitations

This retrospective case series is dependent on the availability and accuracy of available medical records, from two different institutions and suffers from limited generalizability, common with small case series.

## Disclosures

This case report did not receive any funds from funding agencies in the public, commercial, or not-for-profit sectors.

Consent from all patients in the current case series report has been obtained.

## CRediT authorship contribution statement

**Luis Fernandez:** Conceptualization, Data curation, Investigation, Methodology, Project administration, Validation, Visualization, Writing – original draft, Writing – review & editing. **Reginald Carl Baptiste:** Conceptualization, Data curation, Investigation, Methodology, Validation, Writing – review & editing. **Rebekah Bjorklund:** Data curation, Investigation, Methodology, Validation, Writing – review & editing. **Ala'a Alkhatib:** Conceptualization, Data curation, Investigation, Methodology, Validation, Writing – review & editing. **Nesiya Sheriff:** Conceptualization, Data curation, Investigation, Methodology, Validation, Writing – review & editing. **Claudia Sanchez:** Conceptualization, Data curation, Investigation, Methodology, Validation, Writing – review & editing. **Mary Anne Obst:** Conceptualization, Data curation, Investigation, Methodology, Validation, Writing – original draft, Writing – review & editing. **Rebecca Swindall:** Methodology, Validation, Visualization, Writing – review & editing.

## Declaration of competing interest

The authors report no conflicts of interest.
